# Response to: Comment on “Utility of a Novel 12‐Lead ECG Criterion for Localizing Manifest Right‐Sided Accessory Pathways: A Derivation Study”

**DOI:** 10.1002/joa3.70362

**Published:** 2026-05-18

**Authors:** Mahmoud El‐Mowafy, Ahmed Shaaban, Ahmed El‐Damaty

**Affiliations:** ^1^ Department of Cardiac Electrophysiology St Bartholomew's Hospital London UK; ^2^ Cardiac Electrophysiology Section, Cardiovascular Department, Faculty of Medicine Cairo University Cairo Egypt

We sincerely thank the authors for their thoughtful and constructive commentary on our manuscript [1]. Their observations reflect the scientific rigor that drives progress in non‐invasive accessory pathway localization, and we welcome this opportunity to respond to each point raised. We hope the following clarifications and additional context will further strengthen the scientific basis of the PR/QRS ratio and its potential role in pre‐procedural planning for right‐sided accessory pathway ablation [2].

## 1 Derivation Without External Validation

We acknowledge that our study represents a derivation cohort, and that external validation is the natural and necessary next step, one we anticipated and stated clearly in the manuscript's limitations. Importantly, external validation is already underway: we are currently applying the PR/QRS ratio to an independent cohort at Barts Heart Centre, London and Cairo university hospital, Cairo. We expect these results to tell us whether the criterion performs consistently across a different patient population and referral pattern, and to refine the optimal threshold if needed.

## 2 Comparative Benchmarking Against Established Algorithms

We respectfully wish to clarify that comparative benchmarking against established ECG criteria was performed and reported in our manuscript. In the results section, three previously described ECG signs for differentiating right septal from free wall accessory pathways were systematically applied to our patient population [3, 4, 5] with a dedicated comparative analysis presented in figure 9. It is also important to note that the PR/QRS ratio is not intended as a standalone localization algorithm, instead, it is a single focused criterion designed to discriminate between septal and lateral pathway locations, its comparison was therefore appropriately scoped to equivalent criteria within established algorithms, rather than to full multi‐step localization protocols. We hope this clarification places the criterion's purpose and the comparative data in clearer context.

## 3 Sample Characterization and Subgroup Distribution

Subgroup analysis was in fact conducted and reported in our manuscript. Figure 8 shows the distribution of PR, QRS and PR/QRS ratio across septal (AS, MS, PS), intermediate/transition (RA, RP), and free wall (AL, RL, PL). Based on this analysis, intermediate/transition pathways showed a PR/QRS gradient more in keeping with the septal group than the lateral group, which supported our decision to combine them for the primary discriminator analysis, a methodological choice explained in the manuscript. We further tested the hypothesis if there is a similar gradient between anteroseptal (*n* = 6) and posteroseptal (*n* = 30) pathways alone, with the ROC curve available for inclusion should the editorial board consider it a useful addition. Formal statistical comparisons for mid‐septal and right lateral subgroups were intentionally not performed, as both contained only two patients each, too small for any meaningful analysis.

## 4 Inter‐Observer Reproducibility

We wish to clarify that a structured adjudication process was used for all ECG measurements. All PR and QRS interval measurements were performed manually and independently by two electrophysiologists (A.S. and M.E‐M.), with any disagreements referred to a third senior electrophysiologist (A.E‐D.) for final decision. This process was designed to reduce the influence of individual reader variability on the study measurements. We acknowledge that formal intraclass correlation coefficient reporting was not included and agree this would strengthen reproducibility reporting. Prospective ICC analysis between independent readers is planned as part of our external validation study.

## 5 Clinical Applicability and Behavior in Different Patient Groups

We acknowledge this as a valid and clinically relevant concern. How the PR/QRS ratio performs in patients with structural heart disease, significant conduction abnormalities, or other conditions that alter baseline ECG appearances warrants careful consideration. That said, this limitation needs to be seen in the context of the wider patient population; the vast majority of patients with accessory pathways have structurally normal hearts. This naturally reflects both our own cohort and the populations in which most published localization algorithms were developed. Prospective evaluation in patients with coexisting structural disease remains a worthwhile area for future work.

## 6 Conclusion

In summary, we believe the PR/QRS ratio represents a promising, simple, and practical addition to the non‐invasive toolkit for right‐sided accessory pathway localization, one that addresses a real daily challenge in procedural planning and patient counseling. The points raised by the authors are acknowledged and well‐reasoned, and they set a clear agenda for the next phase of this work. External validation in an independent cohort is currently underway, and we look forward to sharing those results with the scientific community in due course.

## Conflicts of Interest

The authors declare no conflicts of interest.

## Data Availability

The data that support the findings of this study are available on request from the corresponding author. The data are not publicly available due to privacy or ethical restrictions.
